# Editorial: Computational Learning Models and Methods Driven by Omics for Precision Medicine

**DOI:** 10.3389/fgene.2020.620976

**Published:** 2020-12-23

**Authors:** Lei Zhu, Hongmin Cai, Fa Zhang, Quan Zou, Yanjie Wei, Huiru Zheng

**Affiliations:** ^1^School of Computer Science and Engineering, South China University of Technology, Guangzhou, China; ^2^Institute of Computing Technology, Chinese Academy of Sciences (CAS), Beijing, China; ^3^Institute of Fundamental and Frontier Sciences, University of Electronic Science and Technology of China, Chengdu, China; ^4^Shenzhen Institutes of Advanced Technology, Chinese Academy of Sciences (CAS), Shenzhen, China; ^5^Faculty of Computing, Engineering and the Built Environment, School of Computing, Engineering and Intelligent Systems, Ulster University, Coleraine, United Kingdom

**Keywords:** omics, machine learning, drug, RNA, disease, biomarker, network analysis, deep learning

Due to the high experimental cost and the exponential decline in the cost of high-throughput sequencing, computational models, and methods are preferred by scholars. The curse of dimensionality is the primary obstacle to dealing with the explosive growth of omics data. Machine learning methods are applied to reduce dimensionality and perform feature selection from massive data. Researchers meet the requirements of data sparsity by increasing the sparsity constraints of the computational models. The models combined with the deep learning method help to discover potential non-linear associations. Improving data representation or adding embedding layers could provide better performance of the models. Computational methods for biomarker discovery, sample classification, and disease process interpretation pave the way for precision medicine.

This topic includes 34 papers and a corrigendum. These papers introduce latest researches in the area of computational biology, catering for precision medicine and complex diseases. They include sequencing alignment, correlation detection between omics data and biological traits, prediction of biological functionality, computational methods for cancer subtyping, finding of pathogenic causes, repositions and targeting, and computational methods specially designed for biological knowledge mining.

## Sequence Alignment

The raw sequencing data is unstructured short sequences. The structured data can be generated from downstream analysis through filtering, quality control, and assembly of these unstructured data. Assembly reconciliation can generate high-quality assembly results. In Tang et al., using the consensus blocks between contigs to construct adjacency graphs to avoid varying sequencing depth and sequencing errors, the authors propose a scoring function to rank the input assembly sets. They use an adjacency algebra model for accurate fusion, which performs well on *M. abscessus, B. fragilis, R. sphaeroides*, and *V. cholerae*. Shi and Zhang apply the partition and recur platform to generate a high-level abstraction of the sequence alignments. The algorithm component library is verified by Apla language. The advantage of implementing the sequence assembly process through abstract components is that it can effectively improve stability and reduce the possibility of errors caused by manual selection.

## Establishing OMICS—Disease Associations

Four groups present research on RNA association prediction, including Long non-coding RNA(lncRNA)–protein interactions (LPI), LncRNA-Disease, microRNA(miRNA)-Disease, and Circular RNA(circRNA)-Disease. Peng et al. give us an overview of how to identify lncRNA–protein interactions(LPI), and they introduced 16 related repositories and methods. Among these network-based and deep learning-based methods for predicting LPI, the proposed SFPEL-LPI used assembly learning and achieved the best Area Under Curve(AUC) performance. Hu et al. combined the two methods of neural network and matrix factorization (MF) to predict lncRNA-disease associations. They achieved this combination by concatenating outputs and sharing inputs between the two methods. Both the MF and the neural network are trained simultaneously under the framework of TensorFlow. In Yu, Shen et al., prior information (lncRNA-miRNA and lncRNA-disease associations) and known miRNA-disease associations are integrated to construct a three-layer heterogeneous network of LncRNA, miRNA, and disease. In this three-layer network, the edges between the layers are filled with prior information. Random walk is applied to predict miRNA-disease associations. The proposed methods are evaluated using cancer data. Their results show that most potential miRNAs can be confirmed by databases. In Lei X. et al., the cirRNA similarity network and the disease similarity network are used as the input of the collaboration filtering recommendation system. Their experiments on predicting potential circRNA–disease associations indicate the effectiveness of the recommendation system algorithm.

Like RNA, microbes and pathogens are also the causes of diseases. In Li, Wang, Chen et al., a bipartite network is applied to avoid the omission of neighbor information for predicting Pathogen–Host associations. Among the top 20 pathogen-host pairs discovered, 16 pairs can be verified by biological experiments. In Ma et al., to explore the pathogenesis of complex diseases from the modular perspective, the similarity matrix is decomposed to generate microbe-disease co-modules by non-negative matrix tri-factorization. Their method achieves nice performance in the enrichment index and the number of significantly enriched taxon sets. In Li S. et al., on the strength of a matrix containing microbes similarity, disease similarity and a bipartite graph network of the two interactions, the potential microbe-disease associations are calculated by Katz centrality. The prediction performance was evaluated by the leave-one-out cross validation and reached an AUC of 0.9098. Zhu et al. use a deep feedforward network to identify microbial markers and realize graph embedding by replacing the first two layers of the network with a sparse graph. Experiments show that this Graph Embedding Deep Feedforward Network has the best performance, comparing deep forest, random forest and Support Vector Machine(SVM).

## Prediction of Biological Functionality

Identifying acetylation proteins is conducive to understanding the post-translational modification process. In Qiu et al., the authors first generate a k-nearest neighbors (KNN) score, and then use random forest to classify the acetylation proteins. The formation of KNN scores is based on domain annotation and subcellular localization. Five-fold cross-validation on the three data sets was performed, and finally, an average AUC of 0.8389 was obtained. In Miao et al., the authors aim to identify which proteins are endoplasmic reticulum-resident proteins, and they achieved accuracy over 86%. Such work allows us to understand the functionality of proteins, which may be potential points of drug design. The promoter drives the flow of genetic information from DNA to RNA, and its sequence information determines the strength of the promoter. In Le et al., the promoter sequence is divided into 10-gram levels and is used to form a 1,000-dimensional vector. The vector is input into a deep neural networks model to classify the promoter strength. Compared with other latest methods in the same test set, this method improves 1–4% on all indicators.

## Computational Approach For Cancer Subtyping

Cancer subtyping is fundamental for precision therapy. Accurately identifying cancer subtypes enables us to understand cancer evolution. In Lu et al., Laplacian score and low-rank representation methods are integrated to obtain a low-rank expression of cancer gene expression data. This low rank matrix is hoping to preserve subtype information. By sorting the obtained matrix, the feature genes are heuristically selected to comprise of a gene subset for accurate cancer subtyping. The method is tested on five cancer dataset and is shown to achieve superior performance over k-means, non-negative matrix factorization (NMF) and several other baseline methods. Aouiche et al. obtained the cancer stages on copy number variation(CNV) data. The positive significance of distinct stage division is dependent on not only a high cure rate after cancer been detected, but also on critical markers, which are potential therapeutic targets. Li, Wang, Wang et al. identify differentially expressed genes(DEGs) in tumor by analyzing the residues of each gene via a regression model and found potential biomarkers of the individual sample from DEGs. Survival analysis is performed on samples collected from human and mouse cancer data, and is shown to be statistically differently.

## Quantitative Understanding of Pathogenic Causes

The goal of developing computational disease models is to find a therapeutic target. As the first step, computational tools are required to explain the cause of the disease. Regarding the identification of Schizophrenia (SZ), Xiang et al. construct a Brainnetome atlas based on resting-state functional magnetic resonance imaging. Brainnetome atlas is a weighted undirected graph constructed with brain regions as nodes and correlations as edges. The authors calculate the features from the altas and, then use least absolute shrinkage and selection operator(lasso) learning to prune the features. The classification is SZ is achieved by using SVM with an accuracy of 93.10%. In Li X. et al., each single sample is classified by a pathway-based approach, into Ulcerative colitis (UC) and Crohn's disease (CD). Even though UC and CD have common clinical characteristics, they have different responses to drugs. According to the gene expression data of the sample, the author scores each pathway to form a pathway activation for single sample matrix, which is classified by a random forest classifier. In Zhang S. et al., the authors aim to select CNV markers to distinguish between three different states of mono-ADP-ribosylhydrolase 2 (MACROD2). The frequent deletions of MACROD2 locus may lead to chromosomal instability of human colorectal cancer. The authors firstly select 17 important single nucleotide polymorphism(SNP) site via mutual information, and then uses bootstrapping scheme to train multiple classifiers. The trained classifiers are finally ensembled to effectively distinguish three types of MACROD2. In Lei W. et al., the effectiveness of lipoprotein 2 on Subarachnoid hemorrhage (SAH) intervention is revealed from the perspective of the cell signaling pathway. The authors discover five biomarkers, three of which have been verified by previous experimental evidence. Finally, the early SAH prediction is performed based on the assembly learning of logistic regression, SVM and Naive-Bayes, achieving an accuracy of 79%. Zhang P. et al. clarify a pathway of polycistronic mRNA ORF73 involved in host apoptosis through protein p53, supplementing the pathogenic process of Kaposi sarcoma-associated herpes virus. This work is mainly done through protein-protein interactions (PPI) analysis, Gene Ontology and Kyoto Encyclopedia of Genes and Genomes pathway analyses. In Shao et al., 108 whole-non-structural protein 5 sequences are analyzed in Zika virus, and 35 potential glycosylation and phosphorylation sites have been discussed. Mutations in amino acid sites are found to be correlated with their pathogenicity and transmission efficiency. The relatively stable nucleic acid sequence is shown to be helpful for detection and vaccine development.

A meta-analysis can combine multiple studies, and the two groups apply meta-analysis methods. In Fukutani et al., after the analysis of Human T-lymphotropic virus 1 (HTLV-1)-infected patients, the authors find that gene CD40LG and gene GBP2 can be used as two phenotypic classifications of HTLV-1 infection, with accuracy rates of 0.88 and 1. In Jin and Shi, a meta-analysis is performed to test SNP-environment interaction. Based on meta-regression (MR), the author proposes overlapping MR combined with the method of processing overlapping data. This method can reduce type I error and is more robust than MR in dealing with the non-linear interaction effect.

Gao et al. screen 107 methylomic features in whole blood methylation samples and use Support Vector Regressor to predict age. What is interesting is that only gene CALB1 and gene KLF14 are both found in the male and female age prediction models.

## Drug Repositions and Targeting

Four works focus on drug repositions. In Manibalan et al., the authors focus on the S100A8 protein, which has a strong interaction with the prevalence of polycystic ovary syndrome biomarkers. Therefore, they design a series of RNA aptamers targeting the S100A8, and select the one with minimal binding energy as the targeted drug. Wound Scratch experiments confirm that the synthesized 18-mer oligo has a significant inhibition effect on tumor cell migration. Wu et al. hope to level the differences in chemotherapy prognosis through cisplatin resistance analysis of oral squamous cell carcinoma. Through the analysis of differentially expressed genes, PPI network and miRNA-mRNA targeted regulatory network, they find that five hub genes and the miR-200 family members that regulate hub genes may be potential drug targets. In Yu, Xu et al., new targeted drugs for hepatocellular carcinoma (HCC) are found by the drug repositioning bioinformatics method. Finding HCC's kernel genes is the first step in work. The next step is to combine the relationship between the drug and gene expression in the Connectivity Map database to score the relationship between the drug and HCC. Among the top ten drugs screened by this method, eight drugs have been supported by publications. In Emdadi and Eslahchi, cell line similarity, drug similarity and half maximal inhibitory concentration are combined to predict the drug sensitivity of cells, and logistic matrix factorization is applied to obtain latent vectors. For the drug sensitivity prediction of the new cell line, the k-nearest neighbors of the cell line are estimated through the decision tree to obtain the latent vectors of the cell line. Finally, a threshold based on the probability of the latent vector is used to predict whether the cell line is sensitive to drugs. The genomics of drug sensitivity on haematopoietic cell lines in cancer was tested for model performance, with an accuracy of 0.721.

## Biology-Oriented Learning Methods

Traditional learning methods have achieved tremendous success and have provided solutions to even some difficult biological problems. In Wang et al., Huber loss is applied to alleviate non-Gaussian noise contaminations. A sparsity penalty item is used to encourage the sparsity of representation of The Cancer Genome Atlas data, and a graph regularization is used to preserve the manifold structure. The clustering accuracy is improved by 5% compared with non-negative matrix factorization. Che et al. improve the traditional methods on the basis of Sparse Group Lasso (SGL) and proposed a weighted sparse group lasso (WSGL) by introducing prior constraint on the sparse term. Compared with lasso and SGL, the performance is significantly improved, indicating that prior biological knowledge carries on valuable message. Comparing the lasso and SGL methods, WSGL can screen less genes, and the ratio of candidate genes is higher using *Arabidopsis* flowering time data. In Lemaçon et al., a visualization method is proposed based on a scoring system for rating susceptibility loci. In general, this is a visualization method for searching for the best potential variants through aggregating prediction approaches. In Guo, Kullback-Leibler divergence is used to measure the distance between two SNPs, and these distances are used as k-means clustering. Then, statistical testing methods are applied to find epistatic interactions, and the time cost of this method is about one-tenth that of Bayesian inference-based method. Zheng et al. use sparse subspace clustering to perform single-cell clustering. This method assumes that the feature vector of a sample can be expressed as a linear combination of other samples in the same subspace. In the test of 10 single-cell datasets, this method maintains the leading position in normalized mutual information and adjusted rand index.

These teams work together to continuously improve model accuracy. Most articles related to computational methods are tailored from early established models for biology knowledge learning.

## Author Contributions

The article was written by LZ, HC, FZ, QZ, YW, and HZ have provided guidance to the manuscript preparation, have also reviewed and edited the paper. All authors have approved the final version of the editorial.

## Conflict of Interest

The authors declare that the research was conducted in the absence of any commercial or financial relationships that could be construed as a potential conflict of interest.

